# Supplementary material for: The causal relationship between the human gut microbiota and pyogenic arthritis: a Mendelian randomization study
Source: Front Cell Infect Microbiol. 2024 Nov 26;14:1452480. doi: 10.3389/fcimb.2024.1452480 (PMC11629706; doi:10.3389/fcimb.2024.1452480)
Supplement: Supplementary file 1 [file DataSheet1.zip › mendelian test/ebi-a-GCST90027840.csv_leave_one_out.pdf]

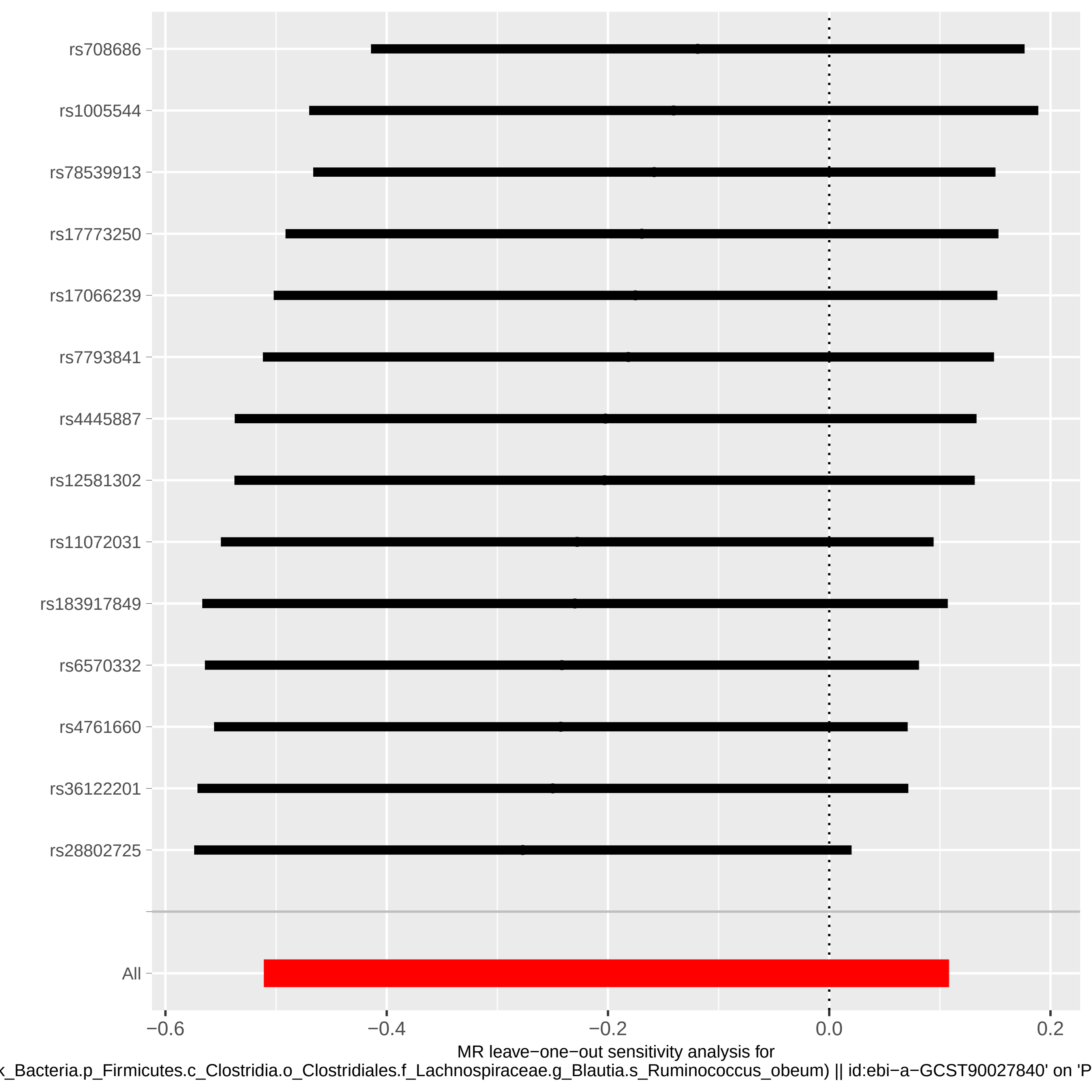

rs708686
rs1005544
rs78539913
rs17773250
rs17066239
rs7793841
rs4445887
rs12581302
rs11072031
rs183917849
rs6570332
rs4761660
rs36122201
rs28802725
All
−0.6
−0.4
−0.2
0.0
0.2
MR leave−one−out sensitivity analysis for
k_Bacteria.p_Firmicutes.c_Clostridia.o_Clostridiales.f_Lachnospiraceae.g_Blautia.s_Ruminococcus_obeum) || id:ebi−a−GCST90027840' on 'P
